# Supplementary material for: Anatomical Registration of Implanted Sensors Improves Accuracy of Trunk Tilt Estimates with a Networked Neuroprosthesis
Source: Sensors (Basel). 2024 Jun 13;24(12):3816. doi: 10.3390/s24123816 (PMC11207283; doi:10.3390/s24123816)
Supplement: Supplementary file 1 [file sensors-24-03816-s001.zip › sensors-3027810-supplementary.pdf]

Kionix®	**± 2g Tri-axis Digital Accelerometer Specifications**	**PART NUMBER:**<br><br>**KXTE9-2050**<br>**Rev. 3**<br>**Nov-2009**

Product Description

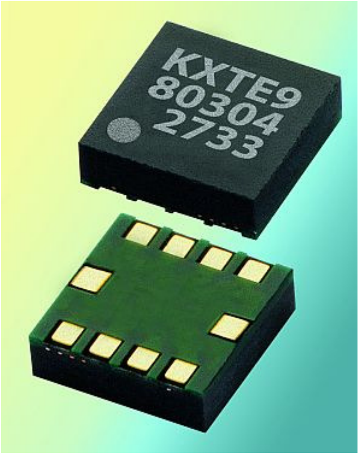

The KXTE9-2050 is a Tri-axis, silicon micromachined accelerometer with a full-scale output range of +/-2g (19.6 m/s/s). The KXTE9 contains integrated orientation and activity detecting algorithms. The sense element is fabricated using Kionix's proprietary plasma micromachining process technology. Acceleration sensing is based on the principle of a differential capacitance arising from acceleration-induced motion of the sense element, which further utilizes common mode cancellation to decrease errors from process variation, temperature, and environmental stress. The sense element is hermetically sealed at the wafer level by bonding a second silicon lid wafer to the device using a glass frit. A separate ASIC device packaged with the sense element provides signal conditioning, digital communication, and embedded logic for orientation and activity detection. The accelerometer is delivered in a 3 x 3 x 0.9 mm LGA plastic package operating from a 1.8 – 3.6V DC supply. An I^2C interface is used for communication with the chip to configure and check updates to the orientation and activity algorithms.

Functional Diagram

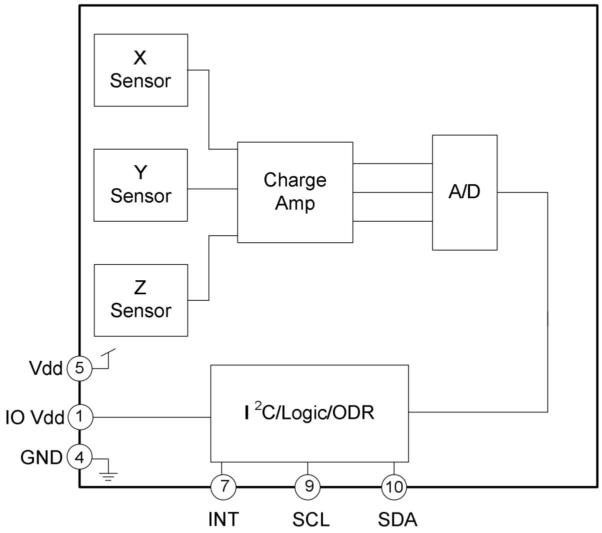

36 Thornwood Dr. – Ithaca, NY 14850
tel: 607-257-1080 – fax:607-257-1146
www.kionix.com - info@kionix.com

© 2009 Kionix – All Rights Reserved

Product Specifications

Table 1. Mechanical

(specifications are for operation at 3.3V and T = 25C unless stated otherwise)

Parameters	Units	Min	Typical	Max
Operating Temperature Range	ºC	-40	-	85
Zero-g Offset	counts		32	
Zero-g Offset Accuracy	mg	-175	0	+175
Zero-g Offset Variation from RT over Temp.	mg/ºC		0.6	
Sensitivity	counts/g		16	
Sensitivity Accuracy	%	-10	0	+10
Sensitivity Variation from RT over Temp.	%/ºC		0.01 (xy)<br>0.03 (z)	
Non-Linearity	% of FS		0.1	
Cross Axis Sensitivity	%		2	
Resolution	mg		62.5	

Table 2. Electrical

(specifications are for operation at 3.3V and T = 25C unless stated otherwise)

Parameters		Units	Min	Typical	Max
Supply Voltage (V_{dd})[1]	Operating	V	1.8	3.3	3.6
I/O Pads Supply Voltage (V_{IO})[1]		V	1.7		V_{dd}
Current Consumption	Operating (full power)	µA	20	30	40
	Standby		-	0.1	
Output Low Voltage[2]		V	-	-	0.3 * V_{io}
Output High Voltage		V	0.9 * V_{io}	-	-
Input Low Voltage		V	-	-	0.2 * V_{io}
Input High Voltage		V	0.8 * V_{io}	-	-
Input Pull-down Current		µA		0	
Power Up Time[3]		ms		50.5	
I^2C Communication Rate		kHz			400
Output Data Rate (ODR)[4]		Hz	1 NaN		40
Bandwidth (-3dB)		Hz		2000	

Notes:

1. Minimum voltage supply of 1.65V can be used over a reduced operating temperature range of 0ºC to 45ºC.
2. Assuming minimum 1.5Kohm I^2C pull-up resistor on SCL and SDA.
3. Power up time is to Vdd = valid and device is in active mode.
4. User selectable through I^2C.

36 Thornwood Dr. – Ithaca, NY 14850
tel: 607-257-1080 – fax:607-257-1146
www.kionix.com - info@kionix.com

© 2009 Kionix – All Rights Reserved

Table 3. Environmental

Parameters		Units	Min	Typical	Max
Supply Voltage (V_{dd})	Absolute Limits	V	-0.3	-	6.0
Operating Temperature Range		ºC	-40	-	85
Storage Temperature Range		ºC	-55	-	150
Mech. Shock (powered and unpowered)		g	-	-	5000 for 0.5ms<br>10000 for 0.2ms
ESD	HBM	V	-	-	2000

Caution: ESD Sensitive and Mechanical Shock Sensitive Component, improper handling can cause permanent damage to the device.

RoHS 2002/95/EC

This product conforms to Directive 2002/95/EC of the European Parliament and of the Council of the European Union (RoHS). Specifically, this product does not contain lead, mercury, cadmium, hexavalent chromium, polybrominated biphenyls (PBB), or polybrominated diphenyl ethers (PBDE) above the maximum concentration values (MCV) by weight in any of its homogenous materials. Homogenous materials are "of uniform composition throughout."

HF

This product is halogen-free per IEC 61249-2-21. Specifically, the materials used in this product contain a maximum total halogen content of 1500 ppm with less than 900-ppm bromine and less than 900-ppm chlorine.

Soldering

Soldering recommendations are available upon request or from www.kionix.com.

36 Thornwood Dr. – Ithaca, NY 14850
tel: 607-257-1080 – fax:607-257-1146
www.kionix.com - info@kionix.com

© 2009 Kionix – All Rights Reserved

Application Schematic

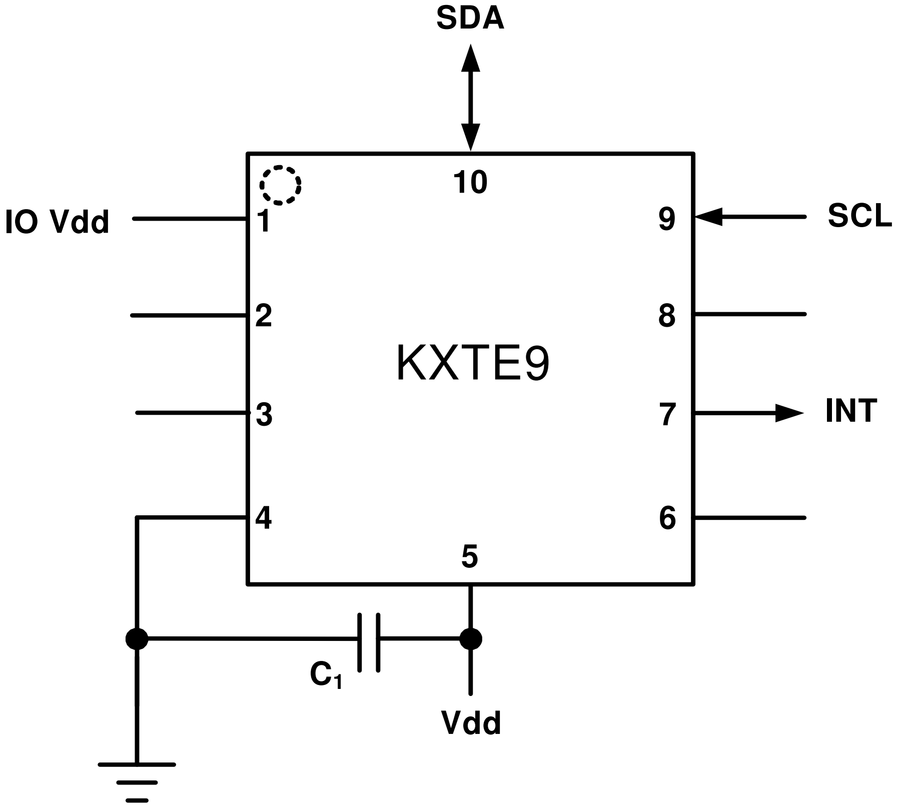

Table 4. KXTE9 Pin Descriptions

Pin	Name	Description
1	IO Vdd	The power supply input for the digital communication bus
2	NC	Not Connected internally – may be connected to Vdd or GND
3	NC	Not Connected internally – may be connected to Vdd or GND
4	GND	Ground
5	Vdd	The power supply input. Decouple this pin to ground with a 0.1uF ceramic capacitor.
6	NC	Not Connected internally – may be connected to Vdd or GND
7	INT	Interrupt pin (Reports user-defined state changes)
8	NC	Not Connected internally – may be connected to Vdd or GND
9	SCL	I^2C Serial Clock (requires 1.5kΩ pull-up resistor)
10	SDA	I^2C Serial Data (requires 1.5kΩ pull-up resistor)

© 2009 Kionix – All Rights Reserved

Test Specifications

! ***Special Characteristics***:

These characteristics have been identified as being critical to the customer. Every part is tested to verify its conformance to specification prior to shipment.

Table 5. Test Specifications

Parameter	Specification	Test Conditions
Zero-g Offset @ RT	32 +/- 2.8 counts	25C, Vdd = 3.3 V
Sensitivity @ RT	16 +/- 1.6 counts/g	25C, Vdd = 3.3 V
Current Consumption -- Operating	20 <= Idd <= 40 uA	25C, Vdd = 3.3 V

36 Thornwood Dr. – Ithaca, NY 14850
tel: 607-257-1080 – fax:607-257-1146
www.kionix.com - info@kionix.com

© 2009 Kionix – All Rights Reserved

Package Dimensions and Orientation

3 x 3 x 0.9 mm LGA

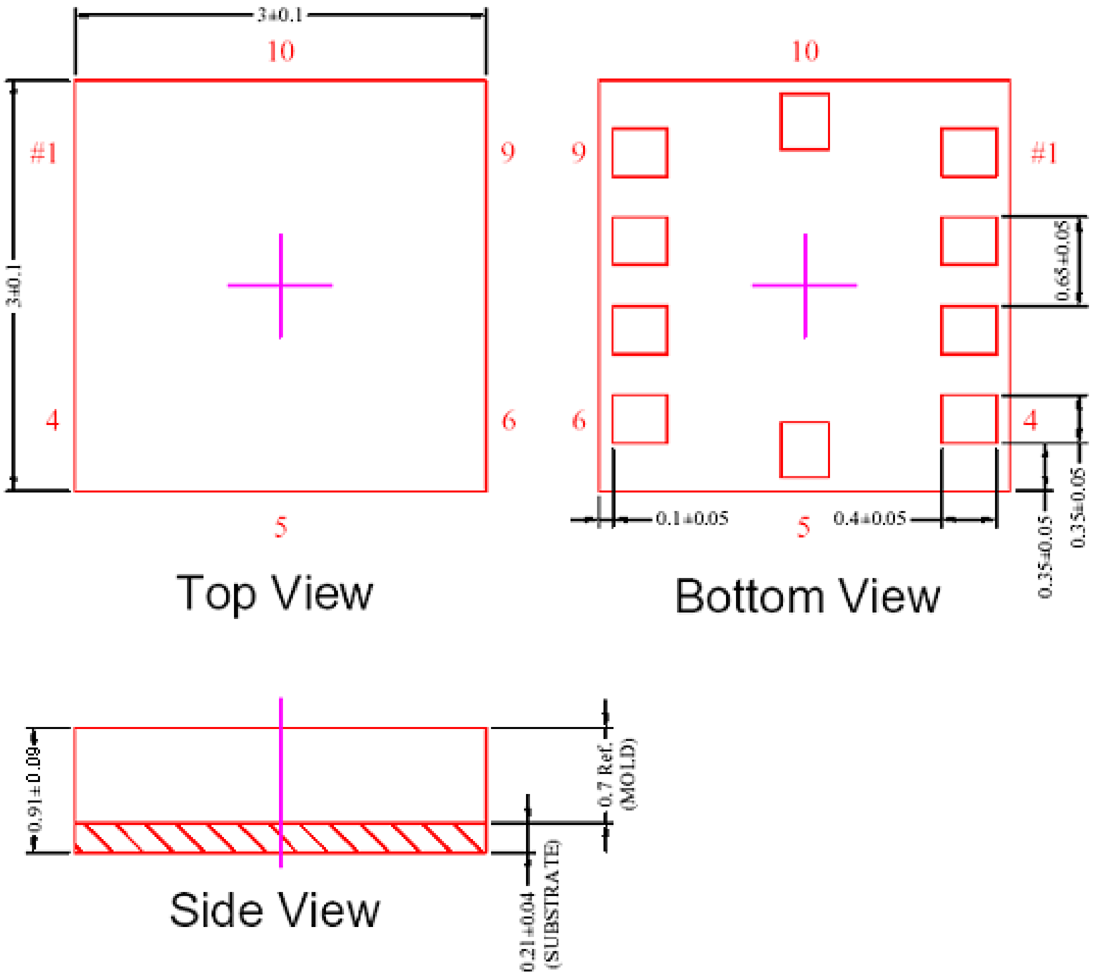

All dimensions and tolerances conform to ASME Y14.5M-1994

Orientation

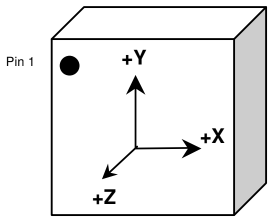

When device is accelerated in +X, +Y or +Z direction, the corresponding output will increase.

Static X/Y/Z Output Response versus Orientation to Earth's surface (1g):

Position	Up State (Y+)	Right State (X+)	Down State (Y-)	Left State (X-)	Face-Up State (Z+)	Face-Down State (Z-)

Position	1	2	3	4	5	6
Diagram	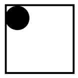	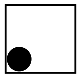	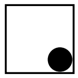	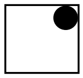	Top<br>Bottom	Bottom<br>Top
X	32 counts	48 counts	32 counts	16 counts	32 counts	32 counts
Y	48 counts	32 counts	16 counts	32 counts	32 counts	32 counts
Z	32 counts	32 counts	32 counts	32 counts	48 counts	16 counts
X-Polarity	0	+	0	-	0	0
Y-Polarity	+	0	-	0	0	0
Z-Polarity	0	0	0	0	+	-

(1-g)

Earth's Surface

© 2009 Kionix – All Rights Reserved

KXTE9 Digital Interface

The Kionix KXTE9 digital accelerometer has the ability to communicate over an I²C digital serial interface bus. This flexibility eases system integration by eliminating analog-to-digital converter requirements and by providing direct communication with system micro-controllers.

The serial interface terms and descriptions indicated in Table 6 below will be observed throughout this document.

Term	Description
Transmitter	The device that transmits data to the bus.
Receiver	The device that receives data from the bus.
Master	The device that initiates a transfer, generates clock signals and terminates a transfer.
Slave	The device addressed by the Master.

Table 6. Serial Interface Terminologies

I²C Serial Interface

The KXTE9 has the ability to communicate on an I²C bus. I²C is primarily used for synchronous serial communication between a Master device and one or more Slave devices. The Master, typically a micro controller, provides the serial clock signal and addresses Slave devices on the bus. The KXTE9 always operates as a Slave device during standard Master-Slave I²C operation.

I²C is a two-wire serial interface that contains a Serial Clock (SCL) line and a Serial Data (SDA) line. SCL is a serial clock that is provided by the Master, but can be held low by any Slave device, putting the Master into a wait condition. SDA is a bi-directional line used to transmit and receive data to and from the interface. Data is transmitted MSB (Most Significant Bit) first in 8-bit per byte format, and the number of bytes transmitted per transfer is unlimited. The I²C bus is considered free when both lines are high.

I²C Operation

Transactions on the I²C bus begin after the Master transmits a start condition (S), which is defined as a high-to-low transition on the data line while the SCL line is held high. The bus is considered busy after this condition. The next byte of data transmitted after the start condition contains the Slave Address (SAD) in the seven MSBs (Most Significant Bits), and the LSB (Least Significant Bit) tells whether the Master will be receiving data '1' from the Slave or transmitting data '0' to the Slave. When a Slave Address is sent, each device on the bus compares the seven MSBs with its internally-stored address. If they match, the device considers itself addressed by the Master. The Slave Address associated with the KXTE9 is **0001111**.

It is mandatory that receiving devices acknowledge (ACK) each transaction. Therefore, the transmitter must release the SDA line during this ACK pulse. The receiver then pulls the data line low so that it remains stable low during the high period of the ACK clock pulse. A receiver that has been addressed, whether it is Master or Slave, is obliged to generate an ACK after each byte of data has been received. To

36 Thornwood Dr. – Ithaca, NY 14850
tel: 607-257-1080 – fax:607-257-1146
www.kionix.com - info@kionix.com

© 2009 Kionix – All Rights Reserved

conclude a transaction, the Master must transmit a stop condition (P) by transitioning the SDA line from low to high while SCL is high. The I^2C bus is now free.

Writing to a KXTE9 8-bit Register

Upon power up, the Master must write to the KXTE9's control registers to set its operational mode. Therefore, when writing to a control register on the I^2C bus, as shown Sequence 1 on the following page, the following protocol must be observed: After a start condition, SAD+W transmission, and the KXTE9 ACK has been returned, an 8-bit Register Address (RA) command is transmitted by the Master. This command is telling the KXTE9 to which 8-bit register the Master will be writing the data. Since this is I^2C mode, the MSB of the RA command should always be zero (0). The KXTE9 acknowledges the RA and the Master transmits the data to be stored in the 8-bit register. The KXTE9 acknowledges that it has received the data and the Master transmits a stop condition (P) to end the data transfer. The data sent to the KXTE9 is now stored in the appropriate register. The KXTE9 automatically increments the received RA commands and, therefore, multiple bytes of data can be written to sequential registers after each Slave ACK as shown in Sequence 2 on the following page.

Reading from a KXTE9 8-bit Register

When reading data from a KXTE9 8-bit register on the I^2C bus, as shown in Sequence 3 on the next page, the following protocol must be observed: The Master first transmits a start condition (S) and the appropriate Slave Address (SAD) with the LSB set at '0' to write. The KXTE9 acknowledges and the Master transmits the 8-bit RA of the register it wants to read. The KXTE9 again acknowledges, and the Master transmits a repeated start condition (Sr). After the repeated start condition, the Master addresses the KXTE9 with a '1' in the LSB (SAD+R) to read from the previously selected register. The Slave then acknowledges and transmits the data from the requested register. The Master does not acknowledge (NACK) it received the transmitted data, but transmits a stop condition to end the data transfer. Note that the KXTE9 automatically increments through its sequential registers, allowing data to be read from multiple registers following a single SAD+R command as shown below in Sequence 4 on the following page.

If a receiver cannot transmit or receive another complete byte of data until it has performed some other function, it can hold SCL low to force the transmitter into a wait state. Data transfer only continues when the receiver is ready for another byte and releases SCL.

36 Thornwood Dr. – Ithaca, NY 14850
tel: 607-257-1080 – fax:607-257-1146
www.kionix.com - info@kionix.com

© 2009 Kionix – All Rights Reserved

Data Transfer Sequences

The following information clearly illustrates the variety of data transfers that can occur on the I^2C bus and how the Master and Slave interact during these transfers. Table 7 defines the I^2C terms used during the data transfers.

Term	Definition
S	Start Condition
Sr	Repeated Start Condition
SAD	Slave Address
W	Write Bit
R	Read Bit
ACK	Acknowledge
NACK	Not Acknowledge
RA	Register Address
Data	Transmitted/Received Data
P	Stop Condition

Table 7. I^2C Terms

Sequence 1. The Master is writing one byte to the Slave.

Master	S	SAD + W		RA		DATA		P
Slave			ACK		ACK		ACK	

Sequence 2. The Master is writing multiple bytes to the Slave.

Master	S	SAD + W		RA		DATA		DATA		P
Slave			ACK		ACK		ACK		ACK	

Sequence 3. The Master is receiving one byte of data from the Slave.

Master	S	SAD + W		RA		Sr	SAD + R			NACK	P
Slave			ACK		ACK			ACK	DATA		

Sequence 4. The Master is receiving multiple bytes of data from the Slave.

Master	S	SAD + W		RA		Sr	SAD + R			ACK		NACK	P
Slave			ACK		ACK			ACK	DATA		DATA		

© 2009 Kionix – All Rights Reserved

KXTE9 Embedded Registers

The KXTE9's 23 embedded 8-bit registers that are accessible via I^2C are listed in Table 8.

Register Name	Type Read/Write	Address Hex	Address Binary
CT_RESP	R	0x0C	0000 1100
Reserved	-	0x0D	0000 1101
Reserved	-	0x0E	0000 1110
WHO_AM_I	R	0x0F	0000 1111
TILT_POS_CUR	R	0x10	0001 0000
TILT_POS_PRE	R	0x11	0001 0001
XOUT	R	0x12	0001 0010
YOUT	R	0x13	0001 0011
ZOUT	R	0x14	0001 0100
Not Used	-	0x15	0001 0101
INT_SRC_REG1	R	0x16	0001 0110
INT_SRC_REG2	R	0x17	0001 0111
STATUS_REG	R	0x18	0001 1000
Not Used	-	0x19	0001 1001
INT_REL	R	0x1A	0001 1010
CTRL_REG1	R/W	0x1B	0001 1011
CTRL_REG2	R/W	0x1C	0001 1100
CTRL_REG3	R/W	0x1D	0001 1101
INT_CTRL_REG1	R/W	0x1E	0001 1110
INT_CTRL_REG2	R/W	0x1F	0001 1111
Not Used	-	0x20 – 0x27	-
TILT_TIMER	R/W	0x28	0010 1000
WUF_TIMER	R/W	0x29	0010 1001
B2S_TIMER	R/W	0x2A	0010 1010
Reserved	-	0x2B – 0x59	-
WUF_THRESH	R/W	0x5A	0101 1010
B2S_THRESH	R/W	0x5B	0101 1011
TILT_ANGLE	R/W	0x5C	0101 1010
Reserved	-	0x5D – 0x5E	-
HYST_SET	R/W	0x5F	0101 1111

Table 8. KXTE9 Register Map

36 Thornwood Dr. – Ithaca, NY 14850
tel: 607-257-1080 – fax:607-257-1146
www.kionix.com - info@kionix.com

© 2009 Kionix – All Rights Reserved

KXTE9 Register Descriptions

CT_RESP

This register has a byte value of 0x55h except when the CTC bit in CTRL_REG3 is set, this value is set to 0xAAh. The byte value is returned to 0x55h after each reading.

R	R	R	R	R	R	R	R	
STR7	STR6	STR5	STR4	STR3	STR2	STR1	STR0	Reset Value
Bit7	Bit6	Bit5	Bit4	Bit3	Bit2	Bit1	Bit0	01010101

I^2C Address: 0x0Ch

WHO_AM_I

This register can be used for supplier recognition, as it can be factory written to a known byte value. The default value is 0x00h.

R	R	R	R	R	R	R	R	
WIA7	WIA6	WIA5	WIA4	WIA3	WIA2	WIA1	WIA0	Reset Value
Bit7	Bit6	Bit5	Bit4	Bit3	Bit2	Bit1	Bit0	00000000

I^2C Address: 0x0Fh

Tilt Position Registers

These two registers report previous and current position data that is updated at the user-defined ODR frequency and is protected during register read. Table 9 describes the reported position for each bit value

TILT_POS_CUR

Current tilt position register

R	R	R	R	R	R	R	R	Reset Value
0	0	LE	RI	DO	UP	FD	FU	00100000
Bit7	Bit6	Bit5	Bit4	Bit3	Bit2	Bit1	Bit0	

I^2C Address: 0x10h

TILT_POS_PRE

Previous tilt position register

R	R	R	R	R	R	R	R	Reset Value
0	0	LE	RI	DO	UP	FD	FU	00100000
Bit7	Bit6	Bit5	Bit4	Bit3	Bit2	Bit1	Bit0	

I^2C Address: 0x11h

36 Thornwood Dr. – Ithaca, NY 14850
tel: 607-257-1080 – fax:607-257-1146
www.kionix.com - info@kionix.com

© 2009 Kionix – All Rights Reserved

Bit	Description
LE	Left State (X-)
RI	Right State (X+)
DO	Down State (Y-)
UP	Up State (Y+)
FD	Face-Down State (Z-)
FU	Face-Up State (Z+)

Table 9. KXTE9 Tilt Position

Accelerometer Outputs

These registers contain 6-bits of valid acceleration data for each axis. The data is updated every ODR period and can be converted from digital counts to acceleration (g) using Equation 1.

Acceleration (g) = (Output (counts) – 0g Offset (counts)) / Sensitivity (counts/g)

Equation 1. Acceleration (g) Calculation

XOUT

X-axis acceleration output (6-bit valid and updated every ODR period)

R	R	R	R	R	R	R	R
XOUTD5	XOUTD4	XOUTD3	XOUTD2	XOUTD1	XOUTD0	X	X
Bit7	Bit6	Bit5	Bit4	Bit3	Bit2	Bit1	Bit0

I^2C Address: 0x12h

YOUT

Y-axis acceleration output (6-bit valid and updated every ODR period)

R	R	R	R	R	R	R	R
YOUTD5	YOUTD4	YOUTD3	YOUTD2	YOUTD1	YOUTD0	X	X
Bit7	Bit6	Bit5	Bit4	Bit3	Bit2	Bit1	Bit0

I^2C Address: 0x13h

ZOUT

Z-axis acceleration output (6-bit valid and updated every ODR period)

R	R	R	R	R	R	R	R
ZOUTD5	ZOUTD4	ZOUTD3	ZOUTD2	ZOUTD1	ZOUTD0	X	X
Bit7	Bit6	Bit5	Bit4	Bit3	Bit2	Bit1	Bit0

I^2C Address: 0x14h

36 Thornwood Dr. – Ithaca, NY 14850
tel: 607-257-1080 – fax:607-257-1146
www.kionix.com - info@kionix.com

© 2009 Kionix – All Rights Reserved

Interrupt Source Registers

These two registers report function state changes. Data may be updated at every ODR period.

INT_SRC_REG1

This register reports which function caused an interrupt.

R	R	R	R	R	R	R	R
0	0	0	0	0	B2SS	WUFS	TPS
Bit7	Bit6	Bit5	Bit4	Bit3	Bit2	Bit1	Bit0

I^2C Address: 0x16h

B2SS *reflects the status of the inactivity/back to sleep function.*

B2SS = 0 – activity state has not changed to inactive

B2SS = 1 – activity state has changed to inactive

WUFS *reflects the status of the activity/wake up function.*

WUFS = 0 – activity state has not changed to active

WUFS = 1 – activity state has changed to active

TPS *reflects the status of the tilt position function.*

TPS = 0 – tilt position state has not changed

TPS = 1 - tilt position state has changed

INT_SRC_REG2

This register reports which axis and direction caused the activity state to transition from inactive to active per Table 10.

R	R	R	R	R	R	R	R
0	0	ALE	ARI	ADO	AUP	AFD	AFU
Bit7	Bit6	Bit5	Bit4	Bit3	Bit2	Bit1	Bit0

I^2C Address: 0x17h

Bit	Description
ALE	X Negative (X-) Reported
ARI	X Positive (X+) Reported
ADO	Y Negative (Y-) Reported
AUP	Y Positive (Y+) Reported
AFD	Z Negative (Z-) Reported
AFU	Z Positive (Z+) Reported

Table 10. KXTE9 Activity Reporting

© 2009 Kionix – All Rights Reserved

STATUS_REG

This register reports the state of the interrupt and the status of information pertaining to the ODR setting.

R	R	R	R	R	R	R	R
0	0	DOR	INT	SODRA	SODRB	0	0
Bit7	Bit6	Bit5	Bit4	Bit3	Bit2	Bit1	Bit0

I^2C Address: 0x18h

***DOR (output data overrun)** bit is released after the next output data register read*
DOR = 0 – no data overrun
DOR = 1 – data is overrun

***INT** reports the combined interrupt information of all enabled functions. This bit is released to 0 when the interrupt release register (1Ah) is read.*
INT = 0 – no interrupt event
INT = 1 – interrupt event has occurred

***SODRA** reports the status of the current ODRA setting that is being used.*
***SODRB** reports the status of the current ODRB setting that is being used.*

INT_REL

Latched interrupt source information is cleared and the physical interrupt latched pin (7) is set to the inactive state when this register is read.

R	R	R	R	R	R	R	R
X	X	X	X	X	X	X	X
Bit7	Bit6	Bit5	Bit4	Bit3	Bit2	Bit1	Bit0

I^2C Address: 0x1Ah

CTRL_REG1

Read/write control register that controls the main feature set.

R/W	R/W	R/W	R/W	R/W	R/W	R/W	R/W	Reset Value
PC1	0	0	ODRA	ODRB	B2SE	WUFE	TPE	00000000
Bit7	Bit6	Bit5	Bit4	Bit3	Bit2	Bit1	Bit0	

I^2C Address: 0x1Bh

***PC1** controls the operating mode of the KXTE9.*
PC1 = 0 - stand-by mode
PC1 = 1 - operating mode

***ODRA** sets the initial output data rate per Table 11.*

© 2009 Kionix – All Rights Reserved

ODRB *sets the initial output data rate per Table 11.*

ODRA	ODRB	Output Data Rate
0	0	1Hz
0	1	3Hz
1	0	10Hz
1	1	40Hz

Table 11. Initial Output Data Rate

B2SE *enables the Back To Sleep function that will detect a transition from active mode to inactive mode.*
B2SE = 0 - disable
B2SE = 1- enable

WUFE *enables the Wake Up function that will detect a transition from inactive mode to active mode.*
WUFE = 0 - disable
WUFE = 1- enable

TPE *enables the Tilt Position function that will detect changes in device orientation.*
TPE = 0 - disable
TPE = 1- enable

CTRL_REG2

Read/write control register that controls tilt position state masking. Per Table 12, if a state's bit is set to one (1), the state change will generate an interrupt. If it is set to zero (0), the state change will not generate an interrupt.

R/W	R/W	R/W	R/W	R/W	R/W	R/W	R/W	Reset Value
0	0	LEM	RIM	DOM	UPM	FDM	FUM	00111111
Bit7	Bit6	Bit5	Bit4	Bit3	Bit2	Bit1	Bit0	

I^2C Address: 0x1Ch

Bit	Description
LEM	Left State Mask
RIM	Right State Mask
DOM	Down State Mask
UPM	Up State Mask
FDM	Face-Down State Mask
FUM	Face-Up State Mask

Table 12. Tilt Position State Mask

36 Thornwood Dr. – Ithaca, NY 14850
tel: 607-257-1080 – fax:607-257-1146
www.kionix.com - info@kionix.com

© 2009 Kionix – All Rights Reserved

CTRL_REG3

Read/write control register that provides more feature set control.

R/W	R/W	R/W	R/W	R/W	R/W	R/W	R/W	Reset Value
SRST	0	0	CTC	OB2SA	OB2SB	OWUFA	OWUFB	00000110
Bit7	Bit6	Bit5	Bit4	Bit3	Bit2	Bit1	Bit0	

I^2C Address: 0x1Dh

SRST *initiates software reset, which returns the internal RAM to Kionix default values.*
This bit will remain high (1) for approximately 50 ms until the RAM load is finished.
SRST = 0 - no action or software reset has finished
SRST = 1 - start software reset

CTC *initiates the communication-test function.*
CTC = 0 - no action
CTC = 1 - sets CT_RESP register to 0xAAh and when CT_RESP is read, sets this bit to 0 and sets CT_RESP to 0x55h

OB2SA *sets the output data rate when in the inactive mode per Table 13.*
OB2SB *sets the output data rate when in the inactive mode per Table 13.*

OB2SA	OB2SB	Output Data Rate
0	0	1Hz
0	1	3Hz
1	0	10Hz
1	1	40Hz

Table 13. Inactive Mode Output Data Rate

OWUFA *sets the output data rate when in the active mode per Table 14.*
OWUFB *sets the output data rate when in the active mode per Table 14.*

OWUFA	OWUFB	Output Data Rate
0	0	1Hz
0	1	3Hz
1	0	10Hz
1	1	40Hz

Table 14. Active Mode Output Data Rate

© 2009 Kionix – All Rights Reserved

INT_CTRL_REG1

This register controls the settings for the physical interrupt pin (7).

R/W	R/W	R/W	R/W	R/W	R/W	R/W	R/W	Reset Value
0	0	0	IEN	IEA	IEL	0	0	00001000
Bit7	Bit6	Bit5	Bit4	Bit3	Bit2	Bit1	Bit0	

I^2C Address: 0x1Eh

IEN *enables/disables the physical interrupt pin (7)*

IEN = 0 - physical interrupt pin (7) is disabled

IEN = 1 - physical interrupt pin (7) is enabled

IEA *sets the polarity of the physical interrupt pin (7)*

IEA = 0 - polarity of the physical interrupt pin (7) is active low

IEA = 1 - polarity of the physical interrupt pin (7) is active high

IEL *sets the response of the physical interrupt pin (7)*

IEL = 0 - the physical interrupt pin (7) latches until it is cleared by reading INT_REL

IEL = 1 - the physical interrupt pin (7) will transmit one pulse with a period of 0.05 ms

INT_CTRL_REG2

This register controls activity/inactivity state masking. Per Table 15, if a state's bit is set to one (1), the state change will generate an interrupt. If it is set to zero (0), the state change will not generate an interrupt.

R/W	R/W	R/W	R/W	R/W	R/W	R/W	R/W	Reset Value
XBW	YBW	ZBW	0	0	0	0	0	11100000
Bit7	Bit6	Bit5	Bit4	Bit3	Bit2	Bit1	Bit0	

I^2C Address: 0x1Fh

Bit	Description
XBW	X-Axis State Mask
YBW	Y-Axis State Mask
ZBW	Z-Axis State Mask

Table 15. Activity/Inactivity State Mask

© 2009 Kionix – All Rights Reserved

TILT_TIMER

This register is the programmable count register for the tilt position state timer (0 to 255 counts). Every count is calculated as 1/ODR delay period. A new state must be valid as many measurement periods before the change is accepted.

R/W	R/W	R/W	R/W	R/W	R/W	R/W	R/W	Reset Value
TSC7	TSC6	TSC5	TSC4	TSC3	TSC2	TSC1	TSC0	00000000
Bit7	Bit6	Bit5	Bit4	Bit3	Bit2	Bit1	Bit0	

I^2C Address: 0x28h

WUF_TIMER

This register is the programmable count register for the inactivity to activity timer (0 to 255 counts). Every count is calculated as 1/ODR delay period. A new state must be valid as many measurement periods before the change is accepted.

R/W	R/W	R/W	R/W	R/W	R/W	R/W	R/W	Reset Value
WUFC7	WUFC6	WUFC5	WUFC4	WUFC3	WUFC2	WUFC1	WUFC0	00000000
Bit7	Bit6	Bit5	Bit4	Bit3	Bit2	Bit1	Bit0	

I^2C Address: 0x29h

B2S_TIMER

This register is the programmable count register for the activity to inactivity timer (0 to 255 counts). Every count is calculated as 16*(1/ODR) delay period. A new state must be valid as many measurement periods before the change is accepted.

R/W	R/W	R/W	R/W	R/W	R/W	R/W	R/W	Reset Value
B2SC7	B2SC6	B2SC5	B2SC4	B2SC3	B2SC2	B2SC1	B2SC0	00000000
Bit7	Bit6	Bit5	Bit4	Bit3	Bit2	Bit1	Bit0	

I^2C Address: 0x2Ah

WUF_THRESH

This register sets the WUF Threshold that is used to detect the transition from inactivity to activity. The KXTE9 ships from the factory with WUF_THRESH set to a change in acceleration of 0.5g.

R/W	R/W	R/W	R/W	R/W	R/W	R/W	R/W	Reset Value
WUFTH7	WUFTH6	WUFTH5	WUFTH4	WUFTH3	WUFTH2	WUFTH1	WUFTH0	00100000
Bit7	Bit6	Bit5	Bit4	Bit3	Bit2	Bit1	Bit0	

I^2C Address: 0x5Ah

36 Thornwood Dr. – Ithaca, NY 14850
tel: 607-257-1080 – fax:607-257-1146
www.kionix.com - info@kionix.com

© 2009 Kionix – All Rights Reserved

B2S_THRESH

This register sets the B2S Threshold that is used to detect the transition from activity to inactivity. The KXTE9 ships from the factory with B2S_THRESH set to a change in acceleration of 1.5g.

R/W	R/W	R/W	R/W	R/W	R/W	R/W	R/W	Reset Value
B2STH7	B2STH6	B2STH5	B2STH4	B2STH3	B2STH2	B2STH1	B2STH0	01100000
Bit7	Bit6	Bit5	Bit4	Bit3	Bit2	Bit1	Bit0	

I^2C Address: 0x5Bh

TILT_ANGLE

This register sets the tilt angle that is used to detect the transition from Face-up/Face-down states to Screen Rotation states. The KXTE9 ships from the factory with tilt angle set to a low threshold of 26° from horizontal. A different default tilt angle can be requested from the factory. Note that the minimum suggested tilt angle is 10°.

R/W	R/W	R/W	R/W	R/W	R/W	R/W	R/W	Reset Value
TA7	TA6	TA5	TA4	TA3	TA2	TA1	TA0	00011100
Bit7	Bit6	Bit5	Bit4	Bit3	Bit2	Bit1	Bit0	

I^2C Address: 0x5Ch

HYST_SET

This register sets the Hysteresis that is placed in between the Screen Rotation states. The KXTE9 ships from the factory with HYST_SET set to +/-15° of hysteresis. A different default hysteresis can be requested from the factory. Note that when writing a new value to this register the current values of RES0 and RES1 must be preserved. These values are set at the factory and must not change.

R/W	R/W	R/W	R/W	R/W	R/W	R/W	R/W	Reset Value
RES1	RES0	0	0	HYST3	HYST2	HYST1	HYST0	--001000
Bit7	Bit6	Bit5	Bit4	Bit3	Bit2	Bit1	Bit0	

I^2C Address: 0x5Fh

36 Thornwood Dr. – Ithaca, NY 14850
tel: 607-257-1080 – fax:607-257-1146
www.kionix.com - info@kionix.com

© 2009 Kionix – All Rights Reserved

KXTE9 Embedded Applications

Orientation Detection Feature

The orientation detection feature of the KXTE9 will report changes in face up, face down, ± vertical and ± horizontal orientation. This intelligent embedded algorithm considers very important factors that provide accurate orientation detection from low cost tri-axis accelerometers. Factors such as: hysteresis, device orientation angle and delay time are described below as these techniques are utilized inside the KXTE9.

Hysteresis

A 45° tilt angle threshold seems like a good choice because it is halfway between 0° and 90°. However, a problem arises when the user holds the device near 45°. Slight vibrations, noise and inherent sensor error will cause the acceleration to go above and below the threshold rapidly and randomly, so the screen will quickly flip back and forth between the 0° and the 90° orientations. This problem is avoided in the KXTE9 by choosing a hysteresis angle. With a ±15° hysteresis angle, the screen will not rotate from 0° to 90° until the device is tilted to 60° (45°+15°). To rotate back to 0°, the user must tilt back to 30° (45°-15°), thus avoiding the screen flipping problem. Table 16 shows the acceleration limits implemented for ±15° of hysteresis in between the four screen rotation states.

Orientation	X Acceleration (g)	Y Acceleration (g)
0°/360°	$-0.5 < a_x < 0.5$	$a_y > 0.866$
90°	$a_x > 0.866$	$-0.5 < a_y < 0.5$
180°	$-0.5 < a_x < 0.5$	$a_y < -0.866$
270°	$a_x < -0.866$	$-0.5 < a_y < 0.5$

Table 16. Acceleration at the four orientations with ±15° of hysteresis

The KXTE9 allows the user to change the amount of hysteresis in between the four screen rotation states. By simply writing to the HYST_SET register, the user can adjust the amount of hysteresis from ±3° to ±30°. The plot in Figure 1 shows the typical amount of hysteresis applied for a given digital count value.

© 2009 Kionix – All Rights Reserved

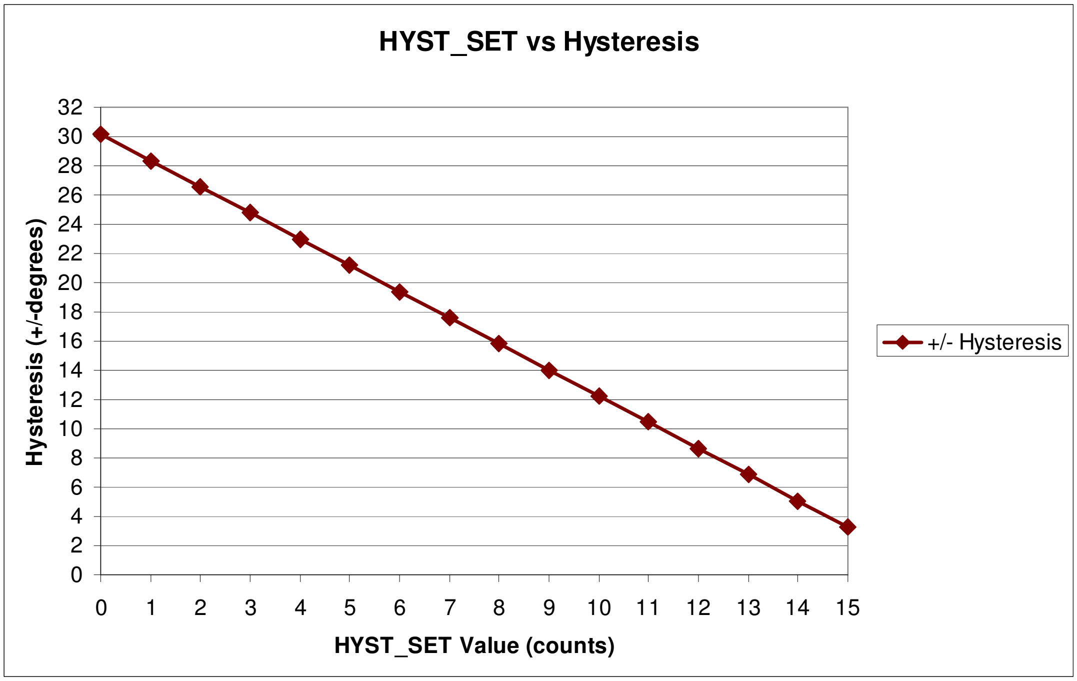

Figure 1. Hysteresis vs. HYST_SET value

Device Orientation Angle (aka Tilt Angle)

To ensure that horizontal and vertical device orientation changes are detected, even when it isn't in the ideal vertical orientation – where the angle θ in Figure 2 is 90°, the KXTE9 considers device orientation angle in its algorithm.

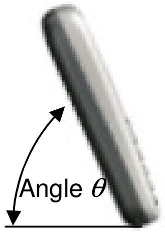

Figure 2. Device Orientation Angle

As the angle in Figure 2 is decreased, the maximum gravitational acceleration on the X-axis or Y-axis will also decrease. Therefore, when the angle becomes small enough, the user will not be able to make the screen orientation change. When the device orientation angle approaches 0° (device is flat on a desk or table), a_x = a_y = 0g, a_z = +1g, and there is no way to determine which way the

36 Thornwood Dr. – Ithaca, NY 14850
tel: 607-257-1080 – fax:607-257-1146
www.kionix.com - info@kionix.com

© 2009 Kionix – All Rights Reserved

screen should be oriented, the internal algorithm determines that the device is in either the face-up or face-down orientation, depending on the sign of the z-axis. The KXTE9 will only change the screen orientation when the orientation angle is above the factory-defaulted/user-defined threshold set in the TILT_ANGLE register. Equation 2 can be used to determine what value to write to the TILT_ANGLE register to set the device orientation angle.

$$\text{TILT\_ANGLE (counts)} = \sin\theta * (\text{Sensitivity (counts/g)} * 4)$$

Equation 2. Device Orientation Angle (aka Tilt Angle)

Tilt Timer

The 8-bit register, TILT_TIMER can be used to qualify changes in orientation. The KXTE9 does this by incrementing a counter with a size that is specified by the value in TILT_TIMER for each set of acceleration samples to verify that a change to a new orientation state is maintained. A user defined output data rate (ODR) determines the time period for each sample. Equation 3 shows how to calculate the TILT_TIMER register value for a desired delay time.

$$\text{TILT\_TIMER (counts)} = \text{Delay Time (sec)} \times \text{ODR (Hz)}$$

Equation 3. Tilt Position Delay Time

36 Thornwood Dr. – Ithaca, NY 14850
tel: 607-257-1080 – fax:607-257-1146
www.kionix.com - info@kionix.com

© 2009 Kionix – All Rights Reserved

Active/Inactive Feature Description

The Active/Inactive feature of the KXTE9 reports qualified changes in acceleration based Wake Up (WUF) and Back to Sleep (B2S) thresholds. If the change in acceleration on any axis is greater than the user-defined wake up threshold (WUF_THRESH), the device has transitioned from an inactive state to an active state. Equation 4 shows how to calculate the WUF_THRESH register value for a desired wake up threshold.

WUF_THRESH (counts) = 4 x (Wake Up Threshold (g) x Sensitivity (counts/g))

Equation 4. Wake Up Threshold

If the change in acceleration on any axis is less than the user-defined back to sleep threshold (B2S_THRESH), the device has transitioned from an active state to an inactive state. Equation 5 shows how to calculate the B2S_THRESH register value for a desired wake up threshold.

B2S_THRESH (counts) = 4 x (Back to Sleep Threshold (g) x Sensitivity (counts/g))

Equation 5. Back to Sleep Threshold

Separate WUF (WUF_TIMER) and B2S (B2S_TIMER) 8-bit raw unsigned values represent counters that permit the user to qualify each active/inactive state change. Note that each WUF Timer count qualifies 1 (one) user-defined ODR period (OB2S) and each B2S Timer count qualifies 16 (sixteen) user-defined periods (initial ODR or OWUF). Equation 6 shows how to calculate the WUF_TIMER register value for a desired wake up delay time.

WUF_TIMER (counts) = Wake Up Delay Time (sec) x OB2S (Hz)

Equation 6. Wake Up Delay Time

Equation 7 shows how to calculate the B2S_TIMER register value for a desired back to sleep delay time.

B2S_TIMER (counts) = (Back to Sleep Delay Time (sec) x OWUF (Hz)) / 16

Equation 7. Back to Sleep Delay Time

Figure 3 shows the response of the Active/Inactive algorithm with WUF Timer = 10 counts and B2S Timer = 10 counts.

© 2009 Kionix – All Rights Reserved

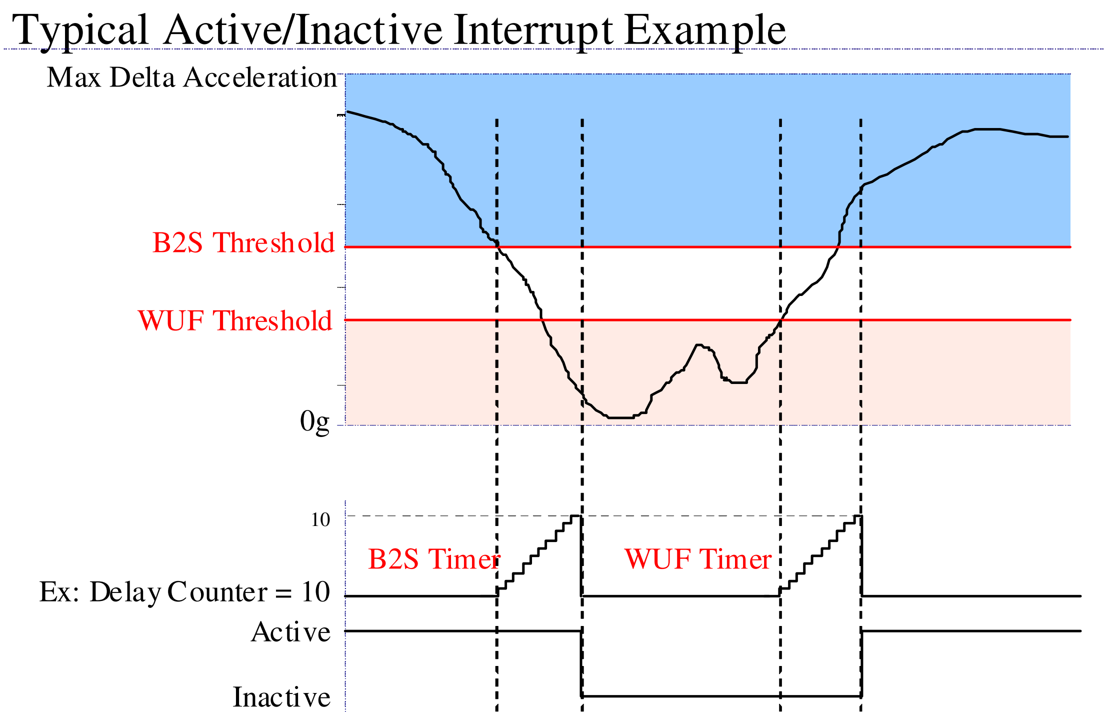

Figure 3. KXTE9 Inactive/Active Transitions

© 2009 Kionix – All Rights Reserved

Revision History

REVISION	DESCRIPTION	DATE
1	Initial release	09-Apr-2009
2	Updated thermal performance	14-Aug-2009
3	Corrected typographical errors and clarified some confusing definitions	05-Nov-2009

"Kionix" is a registered trademark of Kionix, Inc. Products described herein are protected by patents issued or pending. No license is granted by implication or otherwise under any patent or other rights of Kionix. The information contained herein is believed to be accurate and reliable but is not guaranteed. Kionix does not assume responsibility for its use or distribution. Kionix also reserves the right to change product specifications or discontinue this product at any time without prior notice. This publication supersedes and replaces all information previously supplied.

Mouser Electronics

Authorized Distributor

Click to View Pricing, Inventory, Delivery & Lifecycle Information:

Kionix:

EVAL-KXTE9-2050
